# Estimation of amyloid distribution by [^18^F]flutemetamol PET predicts the neuropathological phase of amyloid β-protein deposition

**DOI:** 10.1007/s00401-018-1897-9

**Published:** 2018-08-19

**Authors:** Dietmar Rudolf Thal, Thomas G. Beach, Michelle Zanette, Johan Lilja, Kerstin Heurling, Aruna Chakrabarty, Azzam Ismail, Gill Farrar, Christopher Buckley, Adrian P. L. Smith

**Affiliations:** 1 Department of Neurosciences-Laboratory of Neuropathology, Campus Gasthuisberg (O&N4), KU-Leuven, Herestraat 49, 3000 Leuven, Belgium; 2 Department of Pathology, UZ-Leuven, Leuven, Belgium; 3 Civin Laboratory for Neuropathology, Banner Sun Health Research Institute, Sun City, AZ USA; 4 GE Healthcare Life Sciences, Marlborough, MA USA; 5 GE Healthcare Life Sciences, Uppsala, Sweden; 6 Hermes Medical Solutions AB, Stockholm, Sweden; 7 Department of Psychiatry and Neurochemistry, Wallenberg Centre for Molecular and Translational Medicine, University of Gothenburg, Gothenborg, Sweden; 8 Neuropathology Team, Histopathology Department, Bexley Wing, SJUH, Leeds Teaching Hospitals, NHS Trust, Leeds, UK; 9 GE Healthcare Life Sciences, Amersham, UK

**Keywords:** Amyloid β-protein, Neuropathological staging, Amyloid PET, Imaging, [^18^F]Flutemetamol

## Abstract

The deposition of the amyloid β-protein (Aβ) in senile plaques is one of the histopathological hallmarks of Alzheimer’s disease (AD). Aβ-plaques arise first in neocortical areas and, then, expand into further brain regions in a process described by 5 phases. Since it is possible to identify amyloid pathology with radioactive-labeled tracers by positron emission tomography (PET) the question arises whether it is possible to distinguish the neuropathological Aβ-phases with amyloid PET imaging. To address this question we reassessed 97 cases of the end-of-life study cohort of the phase 3 [^18^F]flutemetamol trial (ClinicalTrials.gov identifiers NCT01165554, and NCT02090855) by combining the standardized uptake value ratios (SUVRs) with pons as reference region for cortical and caudate nucleus-related [^18^F]flutemetamol-retention. We tested them for their prediction of the neuropathological pattern found at autopsy. By defining threshold levels for cortical and caudate nucleus SUVRs we could distinguish different levels of [^18^F]flutemetamol uptake termed PET-Aβ phase estimates. When comparing these PET-Aβ phase estimates with the neuropathological Aβ-phases we found that PET-Aβ phase estimate 0 corresponded with Aβ-phases 0-2, 1 with Aβ-phase 3, 2 with Aβ-phase 4, and 3 with Aβ-phase 5. Classification using the PET-Aβ phase estimates predicted the correct Aβ-phase in 72.16% of the cases studied here. Bootstrap analysis was used to confirm the robustness of the estimates around this association. When allowing a range of ± 1 phase for a given Aβ-phase correct classification was given in 96.91% of the cases. In doing so, we provide a novel method to convert SUVR-levels into PET-Aβ phase estimates that can be easily translated into neuropathological phases of Aβ-deposition. This method allows direct conclusions about the pathological distribution of amyloid plaques (Aβ-phases) in vivo. Accordingly, this method may be ideally suited to detect early preclinical AD-patients, to follow them with disease progression, and to provide a more precise prognosis for them based on the knowledge about the underlying pathological phase of the disease.

**Electronic supplementary material:**

The online version of this article (10.1007/s00401-018-1897-9) contains supplementary material, which is available to authorized users.

## Introduction

Alzheimer’s disease is a major cause of dementia and it is pathologically characterized by amyloid plaques and neurofibrillary tangles (NFTs) [4, 5]. Amyloid plaques are extracellular protein aggregates containing amyloid β-protein (Aβ) [26]. NFTs represent intraneuronal aggregates of the abnormal phosphorylated τ-protein [14]. In the human brain Aβ-plaques arise first in neocortical brain areas before they expand in a second phase into allocortical regions, in phase 3 into the basal ganglia (incl. caudate nucleus), hypothalamus and the thalamus, in phase 4 into the midbrain and the medulla oblongata and, finally, in phase 5 into the cerebellum and the pons [36].

Both, Aβ-plaques and NFTs can be visualized in patients with positron emission tomography (PET) techniques [21, 32]. The amyloid PET-tracers [^18^F]flutemetamol, [^18^F]florbetapir and [^18^F]florbetaben are approved as biomarkers for clinical use to determine the presence or absence of amyloid PET-positivity [9, 30, 31, 41]. Recent research showed for amyloid PET as well as for τ-PET that several stages in the progression of the disease can be described in clinical cohorts [8, 13, 15, 32]. Amyloid or τ-PET-based progression markers, however, are not yet validated for their concordance with regional progression of the histopathological lesions and are not yet approved as progression biomarkers for clinical use.

End-of-life studies have shown that current PET-techniques for the visual assessment of amyloid positivity are less sensitive in comparison with the pathological assessment of these lesions [9, 11, 27, 31, 34]. The analysis of standardized uptake value ratios (SUVRs) already indicated an increase of tracer retention with increasing phases of Aβ-deposition [27, 34] but could not yet identify cutting points to identify distinct neuropathological Aβ-phases by amyloid PET estimation. For τ-PET such end-of-life studies are not yet available. Given the anatomical distribution pattern that describes the different stages of NFT-expansion and the different phases of Aβ-plaque distribution the question arises whether PET regional signals predict postmortem regional histopathology distribution.

To clarify the relationship between [^18^F]flutemetamol amyloid PET imaging-related changes with the neuropathologically determined Aβ-plaque phases [36] we reassessed the [^18^F]flutemetamol phase 3 trial cohort of cases included in an end-of-life study [11, 31, 34], determined SUVRs of the tracer in cortical and subcortical brain regions, and analyzed its relationship to the pathologically determined phases of Aβ-deposition.

## Materials and methods

### Subjects

The subject cohort of 97 cases was included in the [^18^F]flutemetamol efficacy analysis of the GE067-007 (ClinicalTrials.gov identifier: NCT01165554) and GE-067-026 (ClinicalTrials.gov identifier: NCT02090855) phase 3 end-of-life clinical trial and autopsied after death (Suppl. Tab. 1) [6, 17]. The criteria for the selection of these 97 cases from all 106 studied subjects included in the trial were the availability of necessary parameters for this study, i.e., Aβ-phases, comparable measurements for cortical and caudate nucleus tracer retention. For 9 cases caudate SUVRs could not be obtained for the reasons listed in Suppl. Tab. 2. Dementia, defined according to the DSM IV criteria, was noted as present or absent. This was a phase 3 multicenter PET study of [^18^F]flutemetamol injection for detecting brain Aβ. Local institutional review boards or ethics committees approved the study protocol before initiation. All subjects or their legal representatives gave prior written informed consent/assent. Consecutive eligible subjects were ≥ 55 years of age, terminally ill with a life expectancy < 3 years, with general health adequate to undergo study procedures. Patients died of natural causes and no serious adverse events were attributable to [^18^F]flutemetamol injection [11]. Subjects were ineligible if they were pregnant/lactating, had known/suspected structural brain abnormalities, contraindication(s) for PET, known/suspected hypersensitivity/allergy to [^18^F]flutemetamol injection (or any component), or had participated in any clinical study using an investigational product within 30 days of signing consent.

The scan-to-death time intervals ranged between 0 and 846 days (mean 215 days; median 154 days).

### Neuropathology assessments

Brain material received at autopsy and previously used for diagnostic purposes supporting the GE067-007/GE-067-026 phase 3 clinical trials was examined. All brains were formalin-fixed. The brains were cut in coronal slices and screened macroscopically. For histopathological analysis and for assessing the amounts of AD-related amyloid plaques, NFTs, and neuritic plaques, we examined paraffin-embedded tissue including parts of the frontal, parietal, temporal, occipital and entorhinal cortex, the hippocampal formation at the level of the lateral geniculate body, basal ganglia, thalamus, amygdala, midbrain, pons, medulla oblongata, and cerebellum. Paraffin sections of 5 µm thickness from all blocks were stained with hematoxylin & eosin and anti-Aβ antibodies (anti-Aβ; 4G8, SIG-39220, Covance, USA, 1:100, formic acid and heat pretreatment). For neuropathological diagnosis sections were stained with the Bielschowsky silver method and immunohistochemical methods for abnormal phosphorylated τ-protein (anti-human PHF-Tau monoclonal antibody; AT8, Prod. No. MN1020, 1:40, Thermo Scientific, United Kingdom), α-synuclein (anti-α-synuclein monoclonal antibody, Prod. No. NCL-L-ASYN, Lot No. 6005209, 1:40, Leica Microsystems, United Kingdom), and ubiquitin (anti-ubiquitin polyclonal antibody, Prod No. Z0458, 1:400, DakoCytomation, United Kingdom). Primary antibodies were detected with biotinylated secondary antibodies (DakoCytomation E0354, UK) and visualized with the DABMap Kit (Ventana, USA). The phase of Aβ-plaque pathology (Aβ-phase) was assessed after screening the Aβ-stained sections for plaque distribution according to previously published protocols [3, 36]. The neuropathological diagnosis of AD-pathology was performed as recommended [16].

### Clinico-pathological classification of cases

Demented cases with at least intermediate NIA-AA degrees of AD-pathology [16] were considered as symptomatic AD cases; non-demented individuals with AD-pathology were referred to as p-preAD cases [28, 34, 38]. Non-demented cases without AD-pathology were classified as non-AD controls. Non-AD controls included cases with NFT-pathology in the medial temporal lobe termed primary age-related tauopathy (PART) [10]. Patients with non-AD dementia encompassed demented patients with vascular dementia, Lewy body disease (LBD) and frontotemporal lobar degeneration with τ-pathology (FTLD-tau: argyrophilic grain disease, NFT-predominant dementia, Pick’s disease) that did not exhibit intermediate or high degrees of AD-pathology indicating that AD-pathology was presumably not responsible for dementia. NFT-predominant dementia was classified according to the recommended classification for FTLD [25] as a distinct form of FTLD-tau and was, therefore, not considered as PART although there is an overlap between PART cases with dementia and NFT-predominant dementia [10, 19].

### [^18^F]Flutemetamol PET image assessments

Amyloid PET imaging was performed at 12 different imaging sites in the USA and in Europe [31, 34]. Before PET imaging, subjects underwent head CT or magnetic resonance imaging (MRI), unless prior images (obtained within 12 months) were available. [^18^F]Flutemetamol injection was administered intravenously at a dose of 185 or 370 MBq of radioactivity at physician discretion [34]. PET images were acquired in 2-min frames on PET/CT cameras, beginning approximately 90-min post-injection, which was attenuation corrected using CT data. Frame-to-frame motion correction was performed on the dynamic data before the frames were averaged to give a 10–20 min scan. Equipment used to capture images varied across the 12 imaging sites [34]. Most images were reconstructed iteratively to form 128 × 128 axial slices, and a Gaussian post-reconstruction smoothing filter was applied to some to achieve uniform image resolution across sites.

The [^18^F]flutemetamol uptake was measured for six gray matter volumes of interest (VOIs) and adjusted for atrophy manually, covering the anterior cingulate, the prefrontal cortex, the lateral temporal cortex, the parietal cortex, one VOI covering both posterior cingulate and precuneus, and one subcortical VOI in the head of the caudate nucleus [6, 34]. Quantitative SUVR calculations were made using pons as reference region [22]. A global neo- and allocortical, composite SUVR (SUVRcort, Table 1) was calculated from SUVRs obtained from anterior cingulate, prefrontal, lateral temporal, parietal, and posterior cingulate cortex including the precuneus region [39]. The SUVR for the caudate nucleus (SUVRcaud, Table 1) was determined based on VOI measurements of both the left and right caudate nucleus (anterior aspect). The caudate VOIs were drawn on a para-sagittal plane which intersected the thalamus, internal capsule, caudate head and frontal white matter. Image processing and VOI analysis was performed using VOIager 4.0.7 (GE Healthcare, Uppsala, Sweden).

**Table 1:** Determinations of Aβ-related parameters, (a) neuropathological parameters, (b) PET-based parameters

(a) Neuropathological parameter	
Aβ-phase	Represents five stages of the hierarchical expansion of Aβ plaque pathology from the neocortex into further brain regions [36]. Recommended parameter for assessing amyloid plaque pathology in the brain for the neuropathological diagnosis of AD [16].
(b) PET-based parameters	
SUVR	Standardized uptake value ratio (SUVR) representing the retention of the amyloid PET-tracer in a given brain region. In this study pons is used as reference region!
SUVRcort	Combined SUVR describing the amyloid PET-tracer retention in five cortical brain regions: anterior cingulate gyrus, frontal cortex, parietal cortex, lateral temporal cortex, posterior cingulate gyrus plus precuneus. In this study pons is used as reference region!
SUVRcaud	SUVR for amyloid PET-tracer retention measured in the head of the caudate nucleus. In this study pons is used as reference region!
SUVRcort + SUVRcaud	Sum of SUVRcort and SUVRcaud by simple addition of the two SUVRs. In this study pons is used as reference region!
PET amyloid stage	Hierarchical staging system for amyloid PET-based detection of first cortical (stage 1) and second striatal PET-tracer retention (stage 2) [8, 13, 15]. The underlying distribution of Aβ plaques as determined neuropathologically with the Aβ phase cannot be estimated with this parameter.
PET-Aβ phase estimate	Represents a score based on threshold levels of increasing SUVRcort and SUVRcaud that takes the hierarchical spreading of Aβ-pathology into account and that allows the translation of [18F]flutemetamol-based SUVRs into estimated underlying Aβ-phases.

To allow staging of amyloid PET Aβ-pathology progression as suggested by Hanseeuw et al. [15], Grothe et al. [13] and Cho et al. [8] (Table 1), visual interpretation of the PET/CTs was carried out by 5 independent raters, who were experienced in PET analysis. The overall presence/absence of amyloid as well as the presence/absence of striatal amyloid was assessed (Suppl. Tab. 1). Striatal amyloid was, thereby, visually assessed in the head of the caudate nucleus and the putamen [6]. For this procedure with [^18^F]flutemetamol an inter-rater read agreement of at least 80% has been previously published [6, 11, 17]. For determination of the PET-Aβ stages [15] we used the majority reads.

### Statistical analysis

Linear regression analysis was calculated using SPSS 24 statistical software (IBM, Armonk, NY, USA). For this purpose the Aβ-phase, which was determined by neuropathological analysis, was set as the dependent variable and SUVRcort, SUVRcaud, SUVRcort + caud, as well as the stages of anatomical distribution of amyloid PET-tracer retention, called the PET-Aβ stages, and the new parameter identified in this study describing threshold levels that determine estimates for the underlying pathological Aβ-phase, i.e., the PET-Aβ phase estimate (see Table 1), were set as independent variables in linear regression model terms. In a first analysis the linear regression model only included the dependent and one independent variable. In the event that associations were found we used a second model term in which the independent variable was controlled for age, sex, and last scan-to-death interval. In addition to *R*^2^ and *p* values the standardized *β* coefficient was determined.

To clarify whether it is better to use the cortical SUVRs obtained in five different regions separately or just their composite, i.e., SUVRcort, we first analyzed by ANOVA using the Games-Howell post-hoc test to correct for multiple testing whether differences exist among the five cortical SUVRs and the composite SUVRcort in distinguishing between the Aβ-phases. Since there were no major differences in distinguishing between the Aβ-phases among these SUVRs (Suppl. Tab. 3) we decided to continue with SUVRcort to represent cortical Aβ-deposition.

To determine thresholds for distinguishing the different neuropathologically defined Aβ-phases by SUVR-levels we determined the mean, median, variance, and range of SUVRcort and SUVRcaud for each Aβ-phase including Aβ-phase 0. To identify the cutting point between brains without detectable Aβ-plaques and with Aβ-pathology we identified the maximum SUVRcort in Aβ-phase 0 and the maximum SUVRcaud in Aβ-phases 0–2 (i.e. in the absence of plaques in the striatum). These maximum SUVRs for amyloid plaque free cortex and caudate nucleus were rounded to the next decimal place to indicate that all SUVRs equal or higher indicate the presence of amyloid. To distinguish between amyloid phases 1–5 we first carried out an ANOVA analysis corrected for multiple testing with a Games-Howell post-hoc test. In the event that a significant increase in SUVR was found when comparing cases with Aβ-phases 0 and 1, 1 and 2, 2 and 3, 3 and 4, or 4 and 5, we decided to identify thresholds for distinguishing the respective Aβ-phase. For determining these thresholds we calculated the mean of the two respective mean SUVRs, e.g., that of the means from Aβ-phases 1 and 2, 2 and 3, 3 and 4, or 4 and 5, respectively, and rounded it to one decimal place by considering the variances: in the event that the cases of the higher Aβ-phase showed a higher variance in SUVRs than those of the lower Aβ-phase then the threshold was determined by rounding to the lower decimal place whereas in the opposite constellation the threshold was determined by rounding the mean of the mean SUVRs to the higher decimal place.

After applying the thresholds and predicting Aβ-phases by PET the percentage of correctly classified cases was calculated as well as that of cases being correctly classified or classified one phase higher or lower than seen at pathological observation. Since we were not able to distinguish between Aβ-phases 0–2 by PET these three phases were considered as one group for calculating the reclassification. Correlation between the neuropathological Aβ-phase and the PET-Aβ phase was calculated with 95% confidence interval (CI). A bootstrap method was performed to resample the data 1000 times using equal probability unrestricted random sampling with replacement and a 95% CI was calculated as a sensitivity analysis to validate the robustness of the estimates.

## Results

### Relationship between Aβ-phases and cortical and caudate nucleus SUVRs

**Fig. 1:**
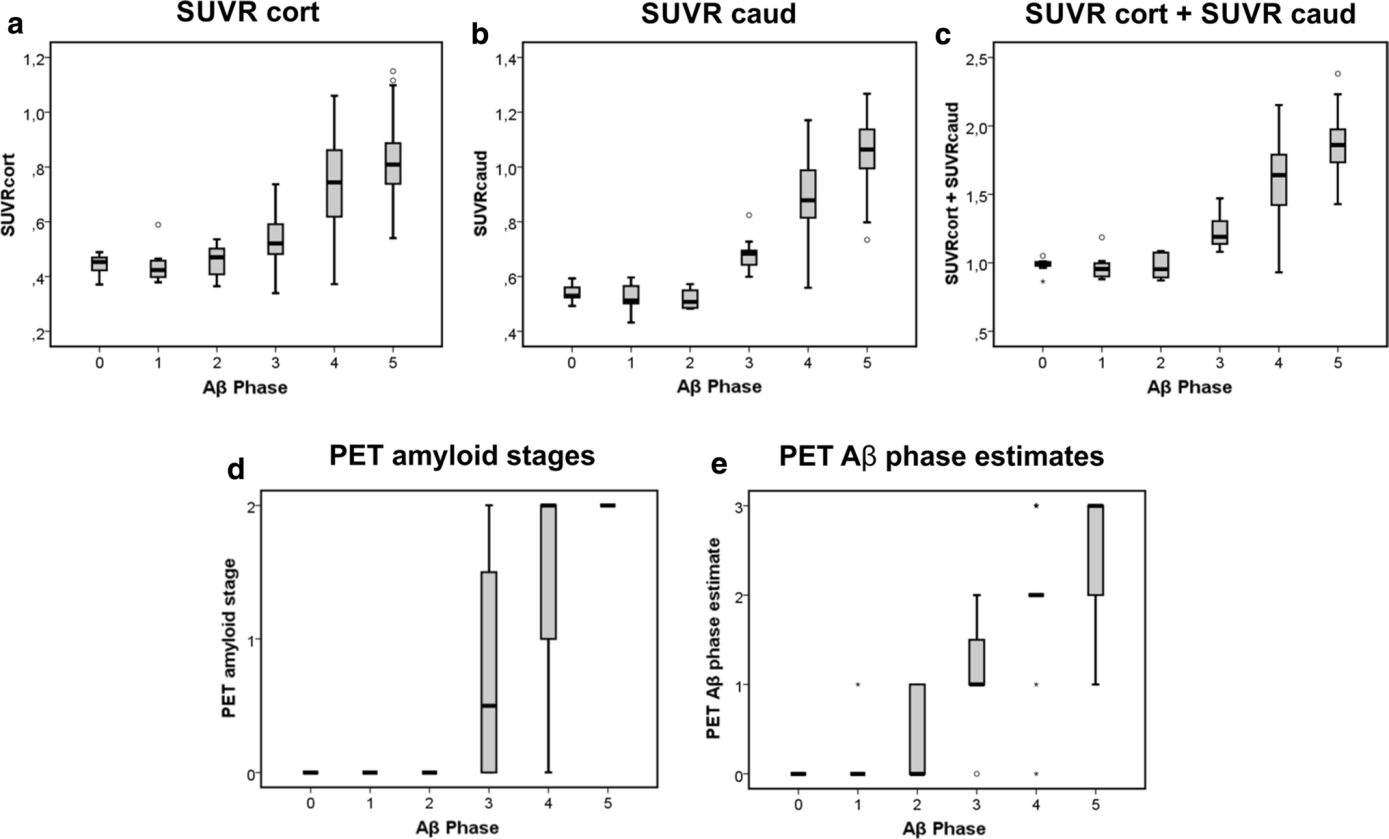
Boxplot diagrams describing the relationship between the pathologically determined Aβ-phases and the cortical SUVR (SUVRcort) (**a**), the caudate nucleus SUVR (SUVRcaud) (**b**), the added cortical and caudate SUVRs (SUVRcort + caud) (**c**), the PET-stage of Aβ-pathology distribution according to Hanseeuw [15] (PET amyloid stage) (**d**), and the PET estimate of the pathological Aβ-phase (PET-Aβ phase estimate) (**e**). Note that the SUVR-based PET-Aβ phase estimates allowed detection of all Aβ-phase 3, 4 and 5 cases as well as of single Aβ-phase 1/2 cases (**e**) whereas Aβ-phase 1, 2 and most Aβ-phase 3 cases were rated as amyloid negative by visual analysis (**d**). The boxes contain the 50% of cases lying in the 2nd and 3rd quartile. The bars indicate the median. The whiskers display the 1.5-times interquartile range. Stars or circles indicate outliers. The individual data of the cases depicted here are provided in Suppl. Tab. 1. *n* = 97 cases

Linear regression analysis of the relationship between the Aβ-phases as the dependent variable and SUVRcort as independent variable revealed an association with *R*^2^ = 0.584 (*p* < 0.001, *β* = 0.764) (Fig. 1a). A model term controlled for age, sex and scan-to-death time interval confirmed this association (*p* < 0.001, *β* = 0.719). A higher *R*^2^ was observed for the correlation between the Aβ-phase as dependent variable and SUVRcaud as independent variable (*R*^2^ = 0.670; *p* < 0.001, *β* = 0.818) (Fig. 1b). This association was also confirmed when controlling the independent variable for age, sex and scan-to-death interval (*p* < 0.001, *β* = 0.771). Combining both cortical and caudate SUVRs by simple addition as independent variable did not increase *R*^2^ indicative for the association between the two parameters (*R*^2^ = 0.666 instead of *R*^2^ = 0.670 for SUVRcaud alone; *p* < 0.001, *β* = 0.816) (Fig. 1c). The same held true for the standardized *β*-coefficient in linear regression analysis controlled for age, sex and scan-to-death interval (*p* < 0.001, *β* = 0.772).

### Relationship between Aβ-phases and PET amyloid staging

The currently published amyloid PET-based staging approaches for Aβ-pathology rely on the measurement/rating of cortical and subcortical (basal ganglia)-related amyloid positivity [8, 13, 15] and the hierarchical involvement of these regions as also known from histopathological studies [36]. Here, we used the majority binary reads (positive/negative) for overall amyloid pathology to define whether there is amyloid in the brain or not. Negative images (those without visible [^18^F]flutemetamol retention) were considered to represent PET amyloid stage 0. Positive images showed at least PET amyloid stage 1 (cortical amyloid deposition). PET amyloid stage 2 was considered when the striatal majority reads indicated amyloid positivity in the striatum as well (Table 1, Suppl. Tab. 1) according to the staging system published by Hanseeuw et al. [15] being compatible with those published by Grothe et al. [13] or Cho et al. [8], which are also based on the subsequent deposition of Aβ first in the cortex and later in the basal ganglia.

Regression analysis between the Aβ-phases as dependent variable and the PET amyloid stages revealed a strong association (*R*^2^ = 0.697, *p* < 0.001, *β* = 0.835) (Fig. 1d), which was confirmed by linear regression analysis controlled for age, sex and scan-to-death interval (*p* < 0.001, *β* = 0.797).

### Distinction of neuropathological Aβ-phases by a multiple threshold-based assessment strategy: PET-Aβ phase estimates

**Fig. 2:**
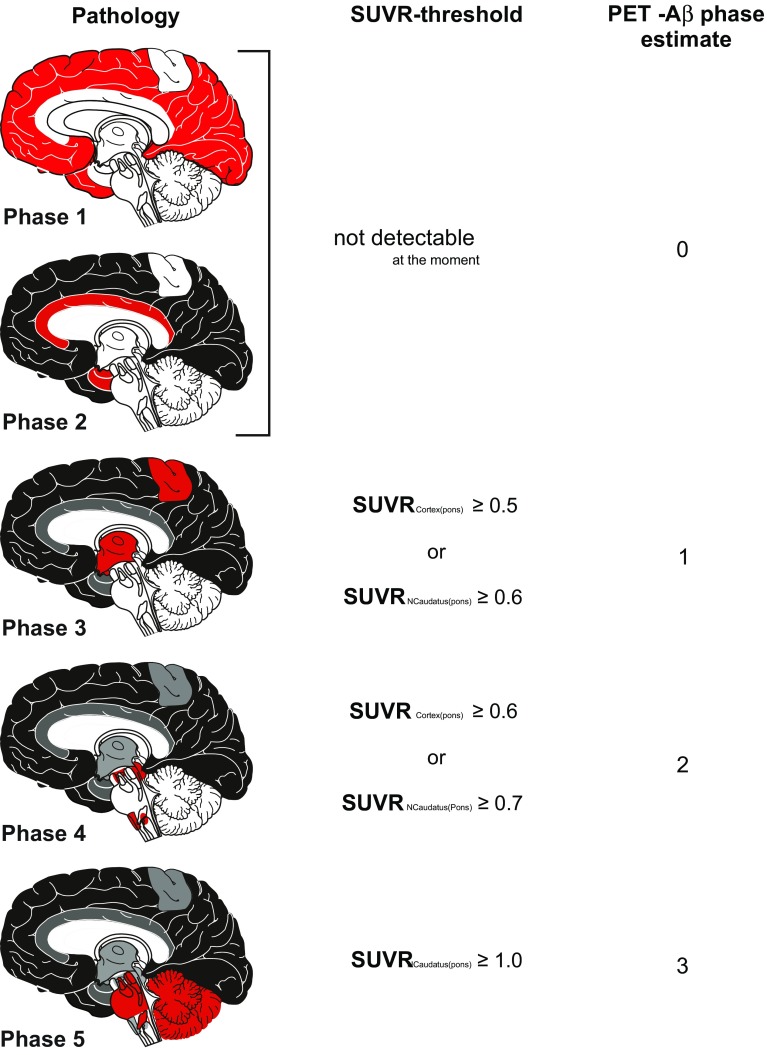
SUVR-based protocol for determination of PET-Aβ phase estimates and its link to the pathologically determined phases of Aβ-plaque deposition [36]. Although Aβ-phases 1 and 2 cannot be detected by [^18^F]flutemetamol PET, cases in Aβ-phase 3 can be identified within one group, i.e. PET-Aβ phase estimate 1, cases in Aβ-phases 4 and 5, respectively, in two further groups, i.e. PET-Aβ phase estimates 2 and 3. The red mark in the schematic representation of the Aβ-phases covers the area in which newly developed plaques in a given phase will develop. This does not mean that the entire red marked field is filled up with Aβ-plaques but that the first small groups of plaques in a given phase of Aβ-depositions can be found there. SUVR_Cortex(pons)_ = SUVRcort; SUVR_NCaudatus(pons)_ = SUVRcaud. Picture elements of this figure were taken from a previously published figure [35] and reused with permission

To identify thresholds between the Aβ-phases we first calculated the range, mean, median, and variance of SUVRcort and SUVRcaud for cases within each Aβ-phase, respectively (Table 2). The range of SUVRcort in cases without Aβ-plaques (= Aβ-phase 0) has been considered as the normal range. The maximum SUVRcort in Aβ-phase 0 cases was 0.489 when using pons as reference region. By rounding to the next decimal place we considered cases with SUVRcort ≥ 0.5 as cases with detectable Aβ-pathology, i.e., Aβ-phase 1 or higher. Likewise, the range of SUVRcaud in cases with Aβ-phases 0–2, i.e., in phases when no plaques were detectable in the striatum, showed a maximum SUVRcaud of 0.596 (using pons as reference region). By rounding to the next decimal place, the threshold for SUVRcaud ≥ 0.6 was set to distinguish cases with and without caudate nucleus Aβ-pathology in addition to the detection of cortical Aβ by SUVRcort. To distinguish the different higher Aβ-phases we compared the mean and median SUVRcorts and SUVRcauds among the Aβ-phases and determined significant differences in the SUVRs of two subsequent Aβ-phases by ANOVA (Table 2). The means of Aβ-phases 0–2 did not vary significantly and were in the range of Aβ-phase 0 cases. Therefore, the first phase that was detected with [18F]flutemetamol amyloid PET was Aβ-phase 3. After phase 3 there was a continuous increase in the mean SUVRcort or SUVRcaud between Aβ-phase 3 and 4 whereas the difference between Aβ-phases 4 and 5 was more pronounced in SUVRcaud than in SUVRcort. The threshold distinguishing Aβ-phase 3 and 4 was oriented on the mean between the means of phase 3 and phase 4 cases. For SUVRcort this mean was 0.64 and for SUVRcaud 0.77. Since the variance of the Aβ-phase 3 SUVRs was lower than that of the Aβ-phase 4 SUVRs we rounded the thresholds to SUVRcort ≥ 0.6 for Aβ-phase 4 and higher and to SUVRcaud ≥ 0.7. The SUVRcort between Aβ-phases 4 and 5 showed major overlap in the ranges (Table 2). Therefore, we did not consider this parameter for the distinction between Aβ-phases 4 and 5. SUVRcaud, on the other hand, showed an obvious difference (Table 2) between Aβ-phases 4 and 5. The mean of the Aβ-phase 4 and 5 means was 0.96. Because the variance of Aβ-phase 5 SUVRcauds was lower than that of Aβ-phase 4 cases the cutting line for the phase 4/5 threshold was rounded up to SUVRcaud ≥ 1. Taken together, the threshold to distinguish Aβ-phases 3 from Aβ-phases 0–2 was either SUVRcort ≥ 0.5 and/or SUVRcaud ≥ 0.6. In comparison to Aβ-phase 3 cases, Aβ-phase 4 cases were best characterized by SUVRcort ≥ 0.6 and/or SUVRcaud ≥ 0.7 and the threshold for Aβ-phase 5 cases was identified as SUVRcaud ≥ 1.0, whereas SUVRcort did not contribute to the distinction between Aβ-phases 4 and 5 (Figs. 1a–e, 2; Table 2). Using these thresholds, four discrete PET-based estimates of the neuropathological Aβ-phase were created, termed PET-Aβ phase estimates (Fig. 2), with PET-Aβ phase estimate 0 = Aβ-phases 0–2, PET-Aβ phase estimate 1 = Aβ-phase 3, PET-Aβ phase estimate 2 = Aβ-phase 4, and PET-Aβ phase estimate 3 = Aβ-phase 5. However, one Aβ-phase 1 and two Aβ-phase 2 cases showed PET-Aβ phase estimate 1 indicating that few Aβ-phase 1 and 2 cases could already be diagnosed as positive for initial plaque pathology with amyloid PET by applying SUVR thresholds, but could not be separated from Aβ-phase 3 cases.

**Table 2:** SUVRcort (a), and SUVRcaud (b): mean, median, range, and variance by Aβ-phase and its comparison by ANOVA with Games-Howell post hoc test

	Mean	Median	Range	Variance	Comparison between phases (ANOVA)	*p*
(a) SUVRcort						
Aβ-phase 0	0.44	0.45	0.37–0.49	0.002		
Aβ-phase 1	0.44	0.44	0.38–0.59	0.004	0 vs. 1	1
Aβ-phase 2	0.46	0.47	0.37–0.54	0.005	1vs. 2	0.999
Aβ-phase 3	0.55	0.54	0.43–0.74	0.009	2 vs. 3	0.280
Aβ-phase 4	0.73	0.74	0.37–1.06	0.028	3 vs. 4	0.005
Aβ-phase 5	0.81	0.80	0.54–1.15	0.013	4 vs. 5	0.415
(b) SUVRcaud						
Aβ-phase 0	0.54	0.53	0.49–0.59	0.001		
Aβ-phase 1	0.51	0.51	0.37–0.59	0.005	0 vs. 1	0.790
Aβ-phase 2	0.52	0.51	0.48–0.57	0.002	1 vs. 2	0.997
Aβ-phase 3	0.66	0.67	0.40–0.82	0.010	2 vs. 3	0.010
Aβ-phase 4	0.88	0.88	0.56–1.17	0.030	3 vs. 4	0.001
Aβ-phase 5	1.04	1.06	0.65–1.27	0.018	4 vs. 5	0.007

Accordingly, we categorized all cases of our sample using SUVRcort and SUVRcaud into PET-Aβ phase estimates. The regression analysis showed a strong association between the PET-Aβ phase estimate as the independent variable and the Aβ-phase as dependent variable (*R*^2^ = 0.759, *p* < 0.001, *β* = 0.871) (Fig. 1e) providing the best relationship between a PET-derived parameter and the real neuropathological distribution of amyloid plaques as described by the Aβ-phases. This finding was in line with that of linear regression analysis controlled for age, sex and scan-to-death interval (*p* < 0.001, *β *= 0.840). The robustness of the estimates in the original study sample was confirmed through the bootstrap analysis. Correlation between PET-Aβ phase and Aβ-phase in the original sample was *r* = 0.88 (95% CI: 0.82, 0.92) and in the bootstrap analysis it was *r* = 0.88 (95% CI: 0.81, 0.92).

### Prediction of pathologically determined Aβ-phases by the PET-Aβ phase estimates

Using the PET-Aβ phase estimate to predict the Aβ-phase based upon PET imaging data sets we could predict the neuropathological phase of Aβ-deposition in 72.16% (70/97 cases) of the cases in our sample. When allowing a range of ± 1 Aβ-phase the Aβ-phase of 96.91% (94/97 cases) of our cases was predicted correctly based upon the PET-Aβ phase estimate. This means in this one-on-one comparison between the pathologically determined Aβ-phases and the PET-Aβ phase estimates that an incorrect prediction of the underlying neuropathological Aβ-phase was in 89% of the incorrect classified cases (24/27) in the range of the neighboring higher or lower Aβ-phase.

Discordances: Regarding the detection of Aβ-phases 1 or 2 it is noteworthy to mention that already 3/14 cases with Aβ-phases 1 or 2 were considered as amyloid positive exhibiting the criteria of PET-Aβ phase estimates 1. Here, one case showed increased cortical SUVRs in all 5 regions, one in 4/5 regions and one in 3/5 regions passing the 0.5 SUVR threshold. In two of these cases with regions with SUVR < 0.5, these PET-negative regions were the anterior cingulate gyrus (1 case), the parietal cortex (1 case) and the lateral temporal cortex (1 case). One phase 3 case did not pass the cortical but only the SUVRcaud threshold whereas the other phase 3 cases showed a heterogeneous distribution of the positive neo- and allocortical regions varying from 1–5 regions passing the cortical SUVR threshold without a distinct predilection site (number of cases with SUVR ≥ 0.5/all Aβ-phase 3 cases: anterior cingulate gyrus: 4/8; frontal cortex: 5/8; parietal cortex: 4/8; lateral temporal cortex: 4/8; posterior cingulate gyrus and precuneus: 6/8). In Aβ-phase 4 four cases showed regional SUVRs under the threshold of amyloid positivity. There were no regions spared of being negative in at least one of these four cases. Two of these cases had Braak NFT-stage I, one Braak NFT-stage II, and one Braak NFT-stage V. From the Aβ-phase 5 cases only one case showed cortical SUVRs lower than 0.5. This was seen in the anterior cingulate gyrus and in the frontal cortex. This case had Braak NFT-stage IV.

## Discussion

This study reports a novel [^18^F]flutemetamol amyloid PET-based method to estimate the anatomical distribution of Aβ-pathology in vivo corresponding to the pathologically assessed Aβ-phases (Fig. 2). This method extends the clinical utility of amyloid PET by allowing a precise estimate of the pathoanatomical distribution of Aβ-plaque pathology in the living patient’s brain even in cases with moderate preclinical AD-pathology which until today could only be determined postmortem. In so doing, this method using the [^18^F]flutemetamol amyloid PET as a biomarker for defined phases of AD-pathology expansion in the brain may be of use in the future for monitoring disease progression and for providing the basis of more valuable prognosis about the expected course of the disease based on its direct link to the underlying pathological phase of the disease. The parameter “PET-Aβ phase estimate”, thereby, serves for translating the SUVR-based thresholds in Aβ-phases as estimated by PET in vivo.

Up to now measurements of increasing Aβ-pathology over time as provided by SUVRs or by applying distinct reading protocols were used to estimate the severity of Aβ-plaque pathology [9, 11, 17, 18, 24, 27, 29, 34] or its progression over time [8, 13, 15]. A precise in vivo estimation of the underlying pathoanatomical plaque distribution, however, was not possible until now although first reports already documented increasing SUVRs with increasing Aβ-phases [27, 34].

Here, estimation of the Aβ-phase was performed by applying distinct thresholds for [^18^F]flutemetamol SUVR in the cortex and caudate nucleus, using the pons as reference region. Thereby, we make use of the hierarchical involvement of the cortex and the caudate nucleus in amyloid plaque deposition as seen pathologically [7, 36] as well as by the increasing levels of all kinds of soluble and insoluble Aβ in the brain [28, 38]. The hierarchical involvement of these two brain regions has also been reported to be visible in amyloid PET imaging and led to the establishment of amyloid PET-based staging systems for Aβ-pathology [8, 13, 15]. However, combining the increase in SUVRs with the expansion of the pathology throughout the brain is a novel approach to predict the extent of amyloid pathology in the brain by amyloid PET. By the use of this strategy we were not only able to distinguish different phases of Aβ-deposition but also to increase the sensitivity of [^18^F]flutemetamol amyloid PET in comparison with the visual assessment of tracer uptake [34]. With our new threshold-based assessment protocol we can now identify more than 90% of the Aβ-phase 3 cases and even single Aβ-phase 1 and 2 cases whereas focusing only on cortical SUVRs allowed to detect only 62% of Aβ-phase 3 cases. Murray et al. [27] reported that even Aβ-phase 1 cases could be identified by amyloid PET but they used a different PET-ligand and a different protocol for the neuropathological assessment of the Aβ-phases, especially a different staining technique, that makes it difficult to compare their results with ours using another presumably more sensitive staining method [1, 2].

That distinct cortical brain regions may represent hot-spots for Aβ-plaque deposition and initial PET-tracer retention can be concluded from the work of Grothe et al. [13] who showed that the temporal lobe and the posterior cingulate gyrus are primarily affected brain regions. One may expect that such effects seen in specific regions may be masked when using the composite SUVR for the cortex. In a pilot analysis we tested whether the regional SUVRs used to calculate the composite SUVR provide more information than the composite SUVRs. Here, we figured out that differences in SUVRs between the distinct Aβ-phases were quite similar among SUVRs assessed in the frontal, parietal, lateral temporal, anterior cingulate and posterior cingulate cortex (plus precuneus) as well as compared with its composite SUVRcort. Therefore, the combination of SUVRcort and SUVRcaud-derived threshold appeared in our hands to represent the most sensitive and straight-forward strategy for estimating the underlying neuropathological Aβ-phase.

The detection of AD-lesions such as that of Aβ-pathology in early preclinical disease stages is of importance because an Aβ-effect on τ-pathology development is considered to occur early in the preclinical phase of AD [23, 33] and until today amyloid PET allowed only the detection of Aβ-pathology in an advanced phase in which its progression already slowed down [23, 34]. Since we detected Aβ histologically in this study with highly sensitive antibodies and determined the phases of Aβ-deposition [34] in 97 cases our findings are difficult to compare with that of other groups who used other amyloid tracers and other methods to detect or quantify Aβ-pathology in smaller cohorts [9, 27, 30]. One group used [^18^F]florbetapir as tracer and quantified Aβ-loads in 59 cases [9], a second group used [^18^F]florbetaben and assessed the plaque densities semi-quantitatively according to the CERAD protocol in 74 cases [30] and the third group used Pittsburg compound B, detected the plaques histopathologically by thioflavin S staining and used these data to determine phases of Aβ-distribution according to a modified protocol in 35 cases [27]. Amongst these studies our neuropathological assessment appears to be, in our opinion, one of the most sensitive and reliable approaches because we used the largest cohort and the 4G8 antibody raised against Aβ_17-24_ for plaque detection. This anti-Aβ_17–24_ antibody has been shown to stain more plaques than other anti-Aβ antibodies, such as those directed against N-terminal epitopes of Aβ [20, 37], and to deliver reliable and sensitive results in inter-laboratory comparison [2]. Moreover, determination of the Aβ-phases, as used here, has been shown to represent a reliable and valid parameter for the assessment of Aβ-plaque pathology [3, 12, 36], which is recommended for the neuropathological assessment of AD-pathology by the National Institute on Aging and the Alzheimer Association [16].

Using the PET-Aβ phase estimation method described here to predict the underlying Aβ-phase in vivo 72.16% of the cases in our sample were correctly classified for the underlying Aβ phase. When allowing a range of ± 1 phase, nearly all cases (96.91%) were appropriately classified. The lack of an independent control collective is a limitation of this study, but our collective of cases is, to our knowledge, the largest end-of-life study cohort used for validation of a PET-ligand detecting amyloid plaques published so far, which required 12 imaging sites to recruit the 97 participants reported here. We decided not to split our cohort into a hypothesis generating and hypothesis confirming group because we wanted to use the maximum power to identify the cut-points. As a sensitivity analysis to assess the robustness of our estimates—given the limitations of an end-of-life study—we used resampling methods based on 1000 bootstrapped samples and were able to confirm the association between Aβ-phase and PET-Aβ phase estimate. Further end-of-life studies on larger collectives in the future could help to validate this method using the methods generated from our sample of 97 cases.

Given the fact that the inter-rater variability for the assessment of amyloid PET images or the neuropathological Aβ-phases is 80% or higher [3, 9, 11, 17] a correct classification of Aβ-phases in 72.2% with a different method looks promising especially since 89% of the incorrect classified cases were classified only one phase higher or lower, i.e., close to the target phase.

A second limitation of this study is that there was unavoidable variation between subjects in the time between the scan and death, the scan-to-death time interval. Thus, we cannot exclude the possibility of changes in amyloid burden between the time of the scan and the postmortem examination of the brain. However, the mean scan-to-death time interval in our study was 215 days which is low in comparison with other studies [9, 27]. Furthermore, we adjusted our statistical analysis appropriately to control not only for age and sex but also for the scan-to-death interval. In addition, prior analyses showed no effect of scan-to-death interval on tracer performance [31].

A third limitation is that we tested only the [^18^F]flutemetamol tracer for PET-Aβ phase estimation. Therefore, we cannot recommend applying [^18^F]flutemetamol thresholds for PET imaging when other tracers are used because of different binding properties to different types of amyloid deposits [22, 40].

## Electronic supplementary material

Below is the link to the electronic supplementary material.

Supplementary material 1 (DOCX 36 kb)

Supplementary material 2 (DOCX 16 kb)
