# Supplementary material for: Estimation of amyloid distribution by [18F]flutemetamol PET predicts the neuropathological phase of amyloid β-protein deposition
Source: Acta Neuropathol. 2018 Aug 19;136(4):557–67. doi: 10.1007/s00401-018-1897-9 (PMC6132944; doi:10.1007/s00401-018-1897-9)
Supplement: Supplementary file 1 — Supplementary material 1 (DOCX 36 kb) [file 401_2018_1897_MOESM1_ESM.docx]

| **Supplementary table 1:** List of cases included in this study | | | | | | |  |  |  |  | |  | |  | |  | |  |  |  |  |  |
| --- | --- | --- | --- | --- | --- | --- | --- | --- | --- | --- | --- | --- | --- | --- | --- | --- | --- | --- | --- | --- | --- | --- |
| **Case Number** | **Age** | **Gender** | **Neuropathological diagnosis** | **Dementia** | **Aβ Phase** | **Braak NFT stage** | **CERAD score** | **NIA-AA-Degree of AD Pathology** | **PET-rating with CT (entire brain)** | | **PET-rating with CT (striatum)** | | **composite SUVRcort-(Neo- and allocortex by pons)** | | **SUVRcaud-(Caudate nucleus by pons)_** | | **PET-amyloid-stage [15]** | | | | **PET-Aβ phase estimate** | **Scan-to-death time interval** |
| **1** | **91** | male | Vascular dementia, Early stage supranuclear palsy | yes | 0 | 0 | 0 | 0 | 0 | | 0 | | 0,40 | | 0,59 | | 0 | | | | 0 | 130 |
| **2** | **66** | male | Ageing changes | yes | 0 | 0 | 0 | 0 | 0 | | 0 | | 0,45 | | 0,56 | | 0 | | | | 0 | 154 |
| **3** | **70** | male | Normal | no | 0 | 0 | 0 | 0 | 0 | | 0 | | 0,49 | | 0,56 | | 0 | | | | 0 | 15 |
| **4** | **63** | male | Normal | no | 0 | 0 | 0 | 0 | 0 | | 0 | | 0,47 | | 0,53 | | 0 | | | | 0 | 12 |
| **5** | **84** | male | PART | yes | 0 | 1 | 0 | 0 | 0 | | 0 | | 0,37 | | 0,49 | | 0 | | | | 0 | 16 |
| **6** | **67** | male | PART | no | 0 | 1 | 0 | 0 | 0 | | 0 | | 0,47 | | 0,53 | | 0 | | | | 0 | 32 |
| **7** | **79** | male | AGD, PART | yes | 0 | 3 | 0 | 0 | 0 | | 0 | | 0,45 | | 0,52 | | 0 | | | | 0 | 130 |
| **8** | **60** | female | Normal | no | 1 | 0 | 0 | 1 | 0 | | 0 | | 0,59 | | 0,59 | | 0 | | | | 1 | 34 |
| **9** | **76** | female | DLB, Vascular dementia | yes | 1 | 2 | 0 | 1 | 0 | | 0 | | 0,41 | | 0,37 | | 0 | | | | 0 | 145 |
| **10** | **62** | male | PSP | no | 1 | 2 | 0 | 1 | 0 | | 0 | | 0,47 | | 0,55 | | 0 | | | | 0 | 432 |
| **11** | **63** | female | Normal | no | 1 | 3 | 0 | 1 | 0 | | 0 | | 0,44 | | 0,51 | | 0 | | | | 0 | 393 |
| **12** | **59** | male | Microinfarcts. | no | 1 | 3 | 0 | 1 | 0 | | 0 | | 0,45 | | 0,52 | | 0 | | | | 0 | 375 |
| **13** | **89** | female | Infarct, Arterolosclerosis | yes | 1 | 3 | 1 | 1 | 0 | | 0 | | 0,38 | | 0,50 | | 0 | | | | 0 | 114 |
| **14** | **82** | female | DLB | yes | 1 | 3 | 1 | 1 | 0 | | 0 | | 0,46 | | 0,43 | | 0 | | | | 0 | 24 |
| **15** | **82** | male | LBD | no | 1 | 4 | 0 | 1 | 0 | | 0 | | 0,40 | | 0,58 | | 0 | | | | 0 | 567 |
| **16** | **72** | female | NFT-predominant dementia | yes | 1 | 5 | 0 | 1 | 0 | | 0 | | 0,41 | | 0,50 | | 0 | | | | 0 | 104 |
| **17** | **74** | male | Multiinfarcts dementia | yes | 2 | 0 | 0 | 1 | 0 | | 0 | | 0,37 | | 0,51 | | 0 | | | | 0 | 169 |
| **18** | **86** | female | Aging changes, Arteriolosclerosis | yes | 2 | 0 | 1 | 1 | 0 | | 0 | | 0,47 | | 0,48 | | 0 | | | | 0 | 136 |
| **19** | **75** | female | Normal | yes | 2 | 1 | 0 | 1 | 0 | | 0 | | 0,54 | | 0,55 | | 0 | | | | 1 | 9 |
| **20** | **84** | female | Vascular Dementia | yes | 2 | 2 | 1 | 1 | 0 | | 0 | | 0,50 | | 0,57 | | 0 | | | | 1 | 68 |
| **21** | **89** | female | AD, CAA (focal) | yes | 2 | 4 | 1 | 1 | 0 | | 0 | | 0,41 | | 0,49 | | 0 | | | | 0 | 77 |
| **22** | **83** | female | CAA. Infarct. Vascular brain injury | no | 3 | 0 | 3 | 1 | 1 | | 1 | | 0,63 | | 0,69 | | 2 | | | | 2 | 189 |
| **23** | **87** | female | Vascular Dementia | yes | 3 | 1 | 1 | 1 | 0 | | 0 | | 0,49 | | 0,69 | | 0 | | | | 1 | 76 |
| **24** | **72** | female | DLB | no | 3 | 1 | 2 | 1 | 1 | | 1 | | 0,59 | | 0,68 | | 2 | | | | 1 | 294 |
| **25** | **92** | male | DLB | no | 3 | 2 | 2 | 1 | 0 | | 0 | | 0,48 | | 0,66 | | 0 | | | | 1 | 322 |
| **26** | **76** | male | DLB | yes | 3 | 2 | 2 | 1 | 1 | | 0 | | 0,74 | | 0,73 | | 1 | | | | 2 | 83 |
| **27** | **75** | male | DLB, Infarcts | yes | 3 | 2 | 2 | 1 | 0 | | 0 | | 0,44 | | 0,64 | | 0 | | | | 1 | 63 |
| **28** | **84** | male | Vascular Dementia | yes | 3 | 2 | 2 | 1 | 1 | | 0 | | 0,59 | | 0,69 | | 1 | | | | 1 | 44 |
| **29** | **92** | female | TDP43 immunopositivity. | yes | 3 | 3 | 1 | 2 | 0 | | 0 | | 0,43 | | 0,40 | | 0 | | | | 0 | 213 |
| **30** | **86** | male | AD | yes | 3 | 3 | 2 | 2 | 0 | | 0 | | 0,50 | | 0,64 | | 0 | | | | 1 | 18 |
| **31** | **81** | female | DLB | yes | 3 | 4 | 2 | 2 | 1 | | 1 | | 0,65 | | 0,82 | | 2 | | | | 2 | 183 |
| **32** | **83** | female | AD, CAA | yes | 3 | 4 | 2 | 2 | 1 | | 0 | | 0,57 | | 0,60 | | 1 | | | | 1 | 747 |
| **33** | **83** | male | AD. LBD. AS. Vascular brain injury | yes | 3 | 5 | 2 | 2 | 0 | | 0 | | 0,49 | | 0,65 | | 0 | | | | 1 | 348 |
| **34** | **81** | male | Normal | no | 4 | 1 | 1 | 1 | 0 | | 0 | | 0,45 | | 0,61 | | 0 | | | | 1 | 189 |
| **35** | **77** | female | AD, LBD | yes | 4 | 1 | 3 | 1 | 1 | | 1 | | 0,69 | | 0,82 | | 2 | | | | 2 | 179 |
| **36** | **81** | female | AS, CAA. Vascular brain injury. TDP43 immunopositivity. | yes | 4 | 1 | 3 | 1 | 0 | | 0 | | 0,56 | | 0,82 | | 0 | | | | 2 | 612 |
| **37** | **60** | male | AD, CAA (focal) | yes | 4 | 2 | 1 | 1 | 0 | | 0 | | 0,37 | | 0,56 | | 0 | | | | 0 | 10 |
| **38** | **89** | male | AD, DLB | yes | 4 | 2 | 2 | 1 | 1 | | 1 | | 0,71 | | 0,82 | | 2 | | | | 2 | 307 |
| **39** | **91** | female | AD, DLB | yes | 4 | 3 | 1 | 2 | 1 | | 1 | | 0,79 | | 0,96 | | 2 | | | | 2 | 131 |
| **40** | **85** | female | AD, DLB | yes | 4 | 3 | 2 | 2 | 1 | | 1 | | 0,66 | | 0,81 | | 2 | | | | 2 | 192 |
| **41** | **79** | male | AD, CAA, metastatic carcinoma | no | 4 | 3 | 2 | 2 | 1 | | 0 | | 0,90 | | 0,75 | | 1 | | | | 2 | 41 |
| **42** | **88** | female | AD, LBD | yes | 4 | 3 | 2 | 2 | 1 | | 0 | | 0,69 | | 0,64 | | 1 | | | | 2 | 267 |
| **43** | **81** | female | AD, DLB | yes | 4 | 4 | 2 | 2 | 1 | | 1 | | 0,99 | | 1,16 | | 2 | | | | 3 | 126 |
| **44** | **87** | male | AD | yes | 4 | 4 | 2 | 2 | 0 | | 0 | | 0,62 | | 0,67 | | 0 | | | | 2 | 22 |
| **45** | **87** | male | AD, CAA | yes | 4 | 4 | 3 | 2 | 1 | | 1 | | 0,82 | | 0,91 | | 2 | | | | 2 | 1 |
| **46** | **91** | male | AD | yes | 4 | 5 | 2 | 3 | 1 | | 1 | | 0,61 | | 0,88 | | 2 | | | | 2 | 29 |
| **47** | **83** | male | AD, LBD | yes | 4 | 5 | 3 | 3 | 1 | | 1 | | 0,83 | | 0,96 | | 2 | | | | 2 | 180 |
| **48** | **80** | male | AD | yes | 4 | 6 | 2 | 3 | 1 | | 1 | | 0,86 | | 1,02 | | 2 | | | | 3 | 276 |
| **49** | **82** | male | AD, CAA | yes | 4 | 6 | 3 | 3 | 1 | | 1 | | 0,74 | | 0,99 | | 2 | | | | 2 | 14 |
| **50** | **83** | female | AD | yes | 4 | 6 | 3 | 3 | 1 | | 1 | | 0,59 | | 0,83 | | 2 | | | | 2 | 198 |
| **51** | **90** | female | AD | yes | 4 | 6 | 3 | 3 | 1 | | 1 | | 0,87 | | 1,17 | | 2 | | | | 3 | 50 |
| **52** | **91** | female | AD, CAA | yes | 4 | 6 | 3 | 3 | 1 | | 1 | | 0,75 | | 0,89 | | 2 | | | | 2 | 55 |
| **53** | **81** | male | AD, DLB, CAA | yes | 4 | 6 | 3 | 3 | 1 | | 1 | | 0,82 | | 1,09 | | 2 | | | | 3 | 204 |
| **54** | **89** | female | AD, CAA, AS, Microinfarcts | yes | 4 | 6 | 3 | 3 | 1 | | 1 | | 1,06 | | 1,05 | | 2 | | | | 3 | 537 |
| **55** | **94** | female | AD | yes | 5 | 3 | 1 | 2 | 1 | | 1 | | 0,74 | | 0,96 | | 2 | | | | 2 | 19 |
| **56** | **88** | female | AD, CAA. Vascular brain injury. Microinfarcts. | yes | 5 | 3 | 1 | 2 | 1 | | 1 | | 1,15 | | 1,23 | | 2 | | | | 3 | 312 |
| **57** | **86** | female | AD, AS. Vascular brain injury. | yes | 5 | 3 | 2 | 2 | 1 | | 1 | | 0,75 | | 1,08 | | 2 | | | | 3 | 318 |
| **58** | **87** | female | DLB, AD | yes | 5 | 4 | 1 | 2 | 1 | | 1 | | 0,74 | | 1,06 | | 2 | | | | 3 | 130 |
| **59** | **74** | male | AD, CAA | yes | 5 | 4 | 2 | 2 | 1 | | 1 | | 0,54 | | 0,65 | | 2 | | | | 1 | 372 |
| **60** | **91** | female | AD | yes | 5 | 4 | 3 | 2 | 1 | | 1 | | 0,87 | | 1,01 | | 2 | | | | 3 | 594 |
| **61** | **86** | male | LBD, CAA | no | 5 | 4 | 3 | 2 | 1 | | 1 | | 0,83 | | 0,89 | | 2 | | | | 2 | 492 |
| **62** | **87** | female | AD | yes | 5 | 5 | 1 | 2 | 1 | | 1 | | 0,79 | | 1,04 | | 2 | | | | 3 | 330 |
| **63** | **83** | female | AD | yes | 5 | 5 | 2 | 3 | 1 | | 1 | | 0,79 | | 1,08 | | 2 | | | | 3 | 193 |
| **64** | **86** | female | AD | yes | 5 | 5 | 3 | 3 | 1 | | 1 | | 0,95 | | 0,96 | | 2 | | | | 2 | 154 |
| **65** | **77** | female | AD, CAA, LBD, AS. Vascular brain injury. Hydrocephalus. | yes | 5 | 5 | 3 | 3 | 1 | | 1 | | 0,81 | | 1,11 | | 2 | | | | 3 | 423 |
| **66** | **93** | female | AD. AS. CAA. LBD. Vascular brain injury. | yes | 5 | 5 | 3 | 3 | 1 | | 1 | | 0,72 | | 1,13 | | 2 | | | | 3 | 201 |
| **67** | **87** | female | AD, CAA, LBD | yes | 5 | 5 | 3 | 3 | 1 | | 1 | | 0,69 | | 0,73 | | 2 | | | | 2 | 768 |
| **68** | **70** | male | AD, CAA | yes | 5 | 5 | 3 | 3 | 1 | | 1 | | 0,92 | | 1,20 | | 2 | | | | 3 | 306 |
| **69** | **80** | male | AD, DLB | yes | 5 | 6 | 1 | 2 | 1 | | 1 | | 0,79 | | 1,05 | | 2 | | | | 3 | 2 |
| **70** | **94** | female | AD | yes | 5 | 6 | 1 | 2 | 1 | | 1 | | 0,77 | | 0,87 | | 2 | | | | 2 | 630 |
| **71** | **72** | female | AD | yes | 5 | 6 | 1 | 2 | 1 | | 1 | | 0,92 | | 1,14 | | 2 | | | | 3 | 549 |
| **72** | **88** | female | AD, DLB | yes | 5 | 6 | 2 | 3 | 1 | | 1 | | 0,66 | | 0,86 | | 2 | | | | 2 | 78 |
| **73** | **78** | male | AD, DLB | yes | 5 | 6 | 2 | 3 | 1 | | 1 | | 0,87 | | 1,16 | | 2 | | | | 3 | 61 |
| **74** | **84** | male | AD, CAA Multiinfarcts | yes | 5 | 6 | 2 | 3 | 1 | | 1 | | 0,96 | | 1,20 | | 2 | | | | 3 | 59 |
| **75** | **95** | female | AD | yes | 5 | 6 | 2 | 3 | 1 | | 1 | | 0,86 | | 1,14 | | 2 | | | | 3 | 14 |
| **76** | **88** | female | AD, DLB, CAA, Arteriolosclerosis | yes | 5 | 6 | 2 | 3 | 1 | | 1 | | 0,87 | | 1,06 | | 2 | | | | 3 | 114 |
| **77** | **71** | male | AD. Microinfarcts. | yes | 5 | 6 | 2 | 3 | 1 | | 1 | | 0,80 | | 1,07 | | 2 | | | | 3 | 267 |
| **78** | **62** | male | AD, AS, SVD | yes | 5 | 6 | 2 | 3 | 1 | | 1 | | 0,69 | | 1,00 | | 2 | | | | 2 | 342 |
| **79** | **93** | female | AD | yes | 5 | 6 | 2 | 3 | 1 | | 1 | | 0,94 | | 1,15 | | 2 | | | | 3 | 243 |
| **80** | **92** | female | AD, CAA, Infarction | yes | 5 | 6 | 2 | 3 | 1 | | 1 | | 0,97 | | 1,21 | | 2 | | | | 3 | 396 |
| **81** | **80** | male | AD | yes | 5 | 6 | 3 | 3 | 1 | | 1 | | 0,67 | | 1,07 | | 2 | | | | 3 | 0 |
| **82** | **72** | male | AD | yes | 5 | 6 | 3 | 3 | 1 | | 1 | | 0,79 | | 1,19 | | 2 | | | | 3 | 1 |
| **83** | **83** | male | AD | yes | 5 | 6 | 3 | 3 | 1 | | 1 | | 0,80 | | 0,99 | | 2 | | | | 2 | 33 |
| **84** | **85** | female | AD | yes | 5 | 6 | 3 | 3 | 1 | | 1 | | 0,89 | | 1,01 | | 2 | | | | 3 | 127 |
| **85** | **80** | male | AD | yes | 5 | 6 | 3 | 3 | 1 | | 1 | | 0,76 | | 1,07 | | 2 | | | | 3 | 170 |
| **86** | **76** | female | AD, CAA | yes | 5 | 6 | 3 | 3 | 1 | | 1 | | 0,68 | | 0,97 | | 2 | | | | 2 | 26 |
| **87** | **65** | female | AD | yes | 5 | 6 | 3 | 3 | 1 | | 1 | | 0,82 | | 1,04 | | 2 | | | | 3 | 139 |
| **88** | **78** | female | AD, DLB | yes | 5 | 6 | 3 | 3 | 1 | | 1 | | 0,70 | | 1,00 | | 2 | | | | 3 | 124 |
| **89** | **73** | female | AD | yes | 5 | 6 | 3 | 3 | 1 | | 1 | | 0,70 | | 1,09 | | 2 | | | | 3 | 26 |
| **90** | **87** | male | AD | yes | 5 | 6 | 3 | 3 | 1 | | 1 | | 0,73 | | 0,82 | | 2 | | | | 2 | 105 |
| **91** | **75** | female | AD | yes | 5 | 6 | 3 | 3 | 1 | | 1 | | 0,89 | | 1,17 | | 2 | | | | 3 | 65 |
| **92** | **82** | male | AD, VD, Microinfarcts | yes | 5 | 6 | 3 | 3 | 1 | | 1 | | 0,89 | | 1,13 | | 2 | | | | 3 | 846 |
| **93** | **84** | female | AD | yes | 5 | 6 | 3 | 3 | 1 | | 1 | | 0,96 | | 1,27 | | 2 | | | | 3 | 435 |
| **94** | **81** | male | AD, CAA, VD, Microinfarcts | yes | 5 | 6 | 3 | 3 | 1 | | 1 | | 0,77 | | 1,17 | | 2 | | | | 3 | 396 |
| **95** | **84** | male | AD, CAA, LBD, Microinfarcts | yes | 5 | 6 | 3 | 3 | 1 | | 1 | | 0,85 | | 0,94 | | 2 | | | | 2 | 768 |
| **96** | **71** | female | AD, CAA, LBD | yes | 5 | 6 | 3 | 3 | 1 | | 1 | | 0,84 | | 1,03 | | 2 | | | | 3 | 564 |
| **97** | **78** | male | AD, CAA, LBD | yes | 5 | 6 | 3 | 3 | 0 | | 1 | | 0,65 | | 0,80 | | 2 | | | | 2 | 333 |

Age in years; AD = Alzheimer’s disease; AGD = argyrophilic grain disease; AS = atherosclerosis of large brain vessels; CAA = cerebral amyloid angiopathy; DLB = dementia with Lewy-bodies; LBD = Lewy body disease; NFT = neurofibrillary tangle; PART = primary age-related tauopathy; PSP = progressive supranuclear palsy; SVD = small vessel disease; TDP43 = transactive response DNA-binding protein; VD = vascular dementia. Scan-to-death time intervals are given as days.

**Supplementary table 2**: List of the 9 excluded cases from the phase 3 Flutemetamol trial and reasons for not including these cases here.

| **Case Number** | **Age** | **Gender** | **Neuropathological diagnosis** | **Dementia** | **Aβ Phase** | **Braak (NFT) stage** | **CERAD score** | **NIA-AA-Degree of AD Pathology** | **Scan-to-death time interval** | **Reason for exclusion** |
| --- | --- | --- | --- | --- | --- | --- | --- | --- | --- | --- |
| Ex 1 | 72 | female | Infarct. TDP43 immunopositivity. | yes | 1 | 2 | 0 | 1 | 360 | Large lesions in the left hemisphere: therefore, we decided to exclude this case |
| Ex 2 | 71 | male | FTLD-tau: Pick’s disease | yes | 3 | 0 | 1 | 1 | 142 | Anterior part of brain low perfusion: neocortical SUVRs could not be properly measured |
| Ex 3 | 91 | female | AD, AS. Vascular brain injury. Microinfarcts. | yes | 3 | 5 | 2 | 2 | 501 | Normal pressure hydrocephalus with very large ventricles: striatum could not be measured |
| Ex 4 | 87 | female | AD, DLB, AS, Infarct | yes | 4 | 2 | 1 | 1 | 118 | Unusual distribution: therefore, we decided to exclude this case |
| Ex 5 | 91 | female | AD, CAA, PD | yes | 4 | 2 | 1 | 1 | 209 | Patient's right hemisphere was compromised: therefore, we decided to exclude this case |
| Ex 6 | 77 | female | AD | yes | 4 | 6 | 3 | 3 | 10 | Excessive uptake in the nasopharynx and high blood retention/poor signal to noise ratio: therefore, we decided to exclude this case |
| Ex 7 | 91 | female | AD | yes | 5 | 4 | 2 | 2 | 756 | Excessive motion: therefore, we decided to exclude this case |
| Ex 8 | 85 | male | AD | yes | 5 | 5 | 2 | 3 | 394 | CT present, but missing PET image: SUVRs could not be measured |
| Ex 9 | 61 | male | AD | yes | 5 | 6 | 3 | 3 | 561 | Low tracer retention in pons: therefore, we decided to exclude this case |

Age in years; AD = Alzheimer’s disease; AS = atherosclerosis of large brain vessels; CAA = cerebral amyloid angiopathy; CT = computed tomography; DLB = dementia with Lewy-bodies; FTLD-tau = frontotemporal lobar degeneration with τ-pathology; PET = positron emission tomography; PD = Parkinson’s disease; SUVR = standardized uptake value ratio; TDP43 = transactive response DNA-binding protein. Scan-to-death time intervals are given as days.
