# Supplementary material for: Estimation of amyloid distribution by [18F]flutemetamol PET predicts the neuropathological phase of amyloid β-protein deposition
Source: Acta Neuropathol. 2018 Aug 19;136(4):557–67. doi: 10.1007/s00401-018-1897-9 (PMC6132944; doi:10.1007/s00401-018-1897-9)
Supplement: Supplementary file 2 — Supplementary material 2 (DOCX 16 kb) [file 401_2018_1897_MOESM2_ESM.docx]

**Supplementary Tab. 3**: Pilot analysis of SUVRs in the five cortical regions (anterior cingulate (SUVRacg), frontal (SUVRfront), parietal (SUVRpar), lateral temporal (SUVRtemp), and posterior cingulate cortex with precuneus (SUVRpcg-prec)) compared with the composite SUVRcort and SUVRcaud: ANOVA with Games-Howell post-hoc test, n = 97 cases.

a. Comparison among Aβ-phases: p values

|  | **SUVRacg** | **SUVRfront** | **SUVRpar** | **SUVRtemp** | **SUVRpcg-prec** | **SUVRcort** | **SUVRcaud** |
| --- | --- | --- | --- | --- | --- | --- | --- |
| Aβ-phase 0 vs. 1 | 0.996 | 0.974 | 0.994 | 0.987 | 1.000 | 1.000 | 0.790 |
| Aβ-phase 1 vs. 2 | 0.999 | 0.999 | 0.995 | 0.923 | 0.999 | 0.999 | 0.997 |
| Aβ-phase 2 vs. 3 | 0.218 | 0.764 | 0.214 | 0.695 | 0.098 | 0.280 | 0.010 |
| Aβ-phase 3 vs. 4 | 0.012 | 0.003 | 0.011 | 0.011 | 0.014 | 0.005 | 0.001 |
| Aβ-phase 4 vs. 5 | 0.597 | 0.385 | 0.533 | 0.484 | 0.443 | 0.415 | 0.007 |

b. Comparison among PET-Aβ phase estimates: p values

|  | **SUVRacg** | **SUVRfront** | **SUVRpar** | **SUVRtemp** | **SUVRpcg-prec** | **SUVRcort** | **SUVRcaud** |
| --- | --- | --- | --- | --- | --- | --- | --- |
| PET-Aβ phase estimate 0 vs. 1 | 0.001 | >0.001 | 0.002 | 0.005 | 0.002 | >0.001 | >0.001 |
| PET-Aβ phase estimate 1 vs. 2 | >0.001 | >0.001 | >0.001 | >0.001 | >0.001 | >0.001 | >0.001 |
| PET-Aβ phase estimate 2 vs. 3 | >0.001 | >0.001 | >0.001 | >0.001 | 0.006 | >0.001 | >0.001 |
